# Pain is what you think: functional magnetic resonance imaging evidence toward a cognitive and affective approach for pain research

**DOI:** 10.3389/fpain.2024.1388460

**Published:** 2024-12-10

**Authors:** Jocelyn M. Powers, Elena Koning, Gabriela Ioachim, Patrick W. Stroman

**Affiliations:** ^1^ Centre for Neuroscience Studies, Queen’s University, Kingston, ON, Canada; ^2^ Department of Biomedical and Molecular Sciences, Queen’s University, Kingston, ON, Canada; ^3^ Department of Physics, Queen’s University, Kingston, ON, Canada

**Keywords:** fMRI, cognitive pain modulation, affective pain modulation, neural connectivity, structural equation modeling, Bayesian regression

## Abstract

The sensory/discriminative domain of pain is often given more consideration than the cognitive and affective influences that ultimately make pain what it is: a highly subjective experience that is based on an individual's life history and experiences. While many investigations of the underlying mechanisms of pain have focused on solely noxious stimuli, few have compared somatosensory stimuli that cross the boundary from innocuous to noxious. Of those that have, there is little consensus on the similarities and differences in neural signaling across these sensory domains. The purpose of this study was to apply our established network connectivity analyses toward the goal of understanding the neural mechanisms behind sensory, cognitive, and affective responses to noxious and innocuous stimuli. Functional MRI data were collected from 19 healthy women and men that experienced warm and hot thermal stimuli across multiple trials. This is a within-subjects cross-sectional experimental study with repeated measures. Ratings of stimulus intensity and unpleasantness that were collected during each run confirmed significant perceptual differences between the two types of stimuli. Despite this finding, no group differences in network connectivity were found across conditions. When individual differences related to pain ratings were investigated, subtle differences were found in connectivity that could be attributed to sensory and association regions in the innocuous condition, and cognitive, affective, and autonomic regions in the pain condition. These results were reflected in the time-course data for each condition. Overall, signaling mechanisms for innocuous and noxious somatosensation are intricately linked, but pain-specific perception appears to be driven by our psychological and autonomic states.

## 1 Introduction

Neuroimaging techniques provide non-invasive tools for studying the neural basis of pain in humans and the complex regulation of nociception. Functional magnetic resonance imaging (fMRI) studies have employed acute noxious stimuli against no stimulus, while others have modulated acute pain with external cognitive/affective tasks to assess descending nociceptive regulation in the brain, brainstem, and spinal cord (1–17). Few fMRI studies, however, have compared noxious and innocuous somatosensory stimuli to determine the basis for pain-specific signaling (7, 18, 19). Pain is known to have a sensory component, related to its location and intensity, and an affective component, related to how unpleasant the experience is. In addition, pain is influenced by cognitive factors such as the expected sensation, whether or not the pain is the focus of attention, and future implications (20–23). Neural activity related to these components of pain is expected to be different when comparing responses to noxious and innocuous stimuli.

A lack of standardization is seen across data acquisition methods, type of stimulus, inclusion of modulators, or analyses, leaving little consensus across the field to identify a “signature” of pain. Significant differences in neural activity between noxious and innocuous stimuli have been found using EEG, EMG, and fMRI, including graded responses to increasing stimulus intensity or aversiveness in regions such as the primary and secondary somatosensory cortices (SI and SII) and insular cortex (IC) (8, 13, 24–29). Some have suggested effects of sex, attention, fear, salience, and autonomic interaction as driving these differences (24, 25, 28, 30). While other investigations have found no significant group differences between stimulus types (8, 13, 29–33).

Researchers have proposed that psychological and autonomic processes are dominant modulators of the perception of noxious and innocuous stimuli, rather than stimulus intensity (30). Results of studies investigating noxious and innocuous stimuli have often found differences relating to affective valences (24), attention to the stimulus or stimulus aversiveness (25, 33), heart rate (32), or the act of rating stimuli (30), but not the stimuli themselves. This highlights the importance of psychological and interoceptive states when processing somatosensory stimuli, and supports a salience detection system for sensory perception (28, 34–36).

The objective of this study was to investigate differences in signaling across networks of regions spanning the central nervous system. We hypothesized that connectivity across a pre-determined network of regions would significantly differ between noxious and innocuous stimuli, indicating an effect specific to the experience of pain. Moreover, we believed that noxious stimulation would produce stronger blood oxygenation-level dependent (BOLD) signal changes in sensory regions and those involved with modulation of pain and descending pain regulation. This expectation was based on the fact that noxious heat stimuli are at higher temperatures and evoke stronger sensations compared to innocuous warm stimuli. Findings from this investigation will provide insight into the complex nature of pain in humans and contribute to a deeper understanding of central somatosensory processing.

## 2 Methods

This study was part of a larger project which included separate brain and brainstem/spinal cord imaging sessions; the research contained in this paper concerns the brain data. All procedures were approved by the institutional human research ethics review board and complied with the Tri-Council Policy Statement on Ethical Conduct for Research Involving Humans. Data were collected between September 2020 and March 2021 during the COVID-19 pandemic, and additional safety precautions were implemented including reduced study personnel, masks, face shields, and increased sanitation of premises. Informed consent for all study procedures was obtained in writing prior to the onset of the study and participants were explicitly informed that they could cease participation at any time.

### 2.1 Participants

Healthy participants were recruited from the local community through online advertisements. Recruitment materials informed individuals that eligible participation would include one training session (described below) and one or two imaging sessions (brain and/or brainstem/spinal cord), which were randomized to avoid order effects. Twenty-two healthy adults (10 female, 12 male) ranging from 19 to 39 years of age (mean 25 ± 7 years) were recruited. Participants were free of any history of neurological disease or injury, major medical illness, psychiatric disorder, or pre-existing pain condition, and were not taking centrally acting medications or prescription medication for pain relief. Individuals were not considered if they had any contraindications for the MRI environment including pregnancy, claustrophobia, metal implants or injuries from metal fragments, or an inability to lie still. Participants were screened for eligibility through a secure online form on the lab website. Three participants chose not to return to the study after the first imaging session, one female after the brainstem/spinal cord session, and two males after the brain session. As a result, brain fMRI data were acquired from 9 females and 12 males. One of the male participants was unable to distinguish between noxious and innocuous stimuli based on ratings from numerical pain scales; therefore, his data were excluded from the analyses. For the same reason, data from one female participant who completed both imaging sessions were excluded from analyses. In total, data from 8 females and 11 males were used for the following analyses (*n* = 19).

Eligible participants were asked to complete a battery of validated questionnaires to characterize individual traits of mental health, social behaviours, and pain catastrophizing, as they relate to the sensory and affective dimensions of pain. The questionnaires included the Beck Depression Inventory-II (BDI-II) (37), the State/Trait Anxiety Inventory (STAI) (38), the Social-Desirability Scale (SDS) (39), and the Pain Catastrophizing Scale (PCS) (40). The BDI-II assesses the affective, motivational, cognitive and somatic symptoms of depression. The STAI measures the transient condition of state anxiety as well as the chronic condition of trait anxiety. The SDS provides an assessment of whether participants are concerned with social approval, such as providing pain ratings in a way that would gain the approval of the researchers. The PCS reflects how individuals respond to pain, such as tendencies to feel helpless and/or magnify the threat value of a stimulus. The resulting scores were used in correlational analyses with pain ratings to determine if personal behavioural characteristics relate to the experience of pain. Individuals were not excluded from participation given high or low scores on any questionnaire, a range of scores were seen on all scales. One participant failed to turn in their completed questionnaires, therefore only 18 completed sets of questionnaires were used for correlational analyses.

### 2.2 Experimental procedures

#### 2.2.1 Protocol training session

**Figure 1:**
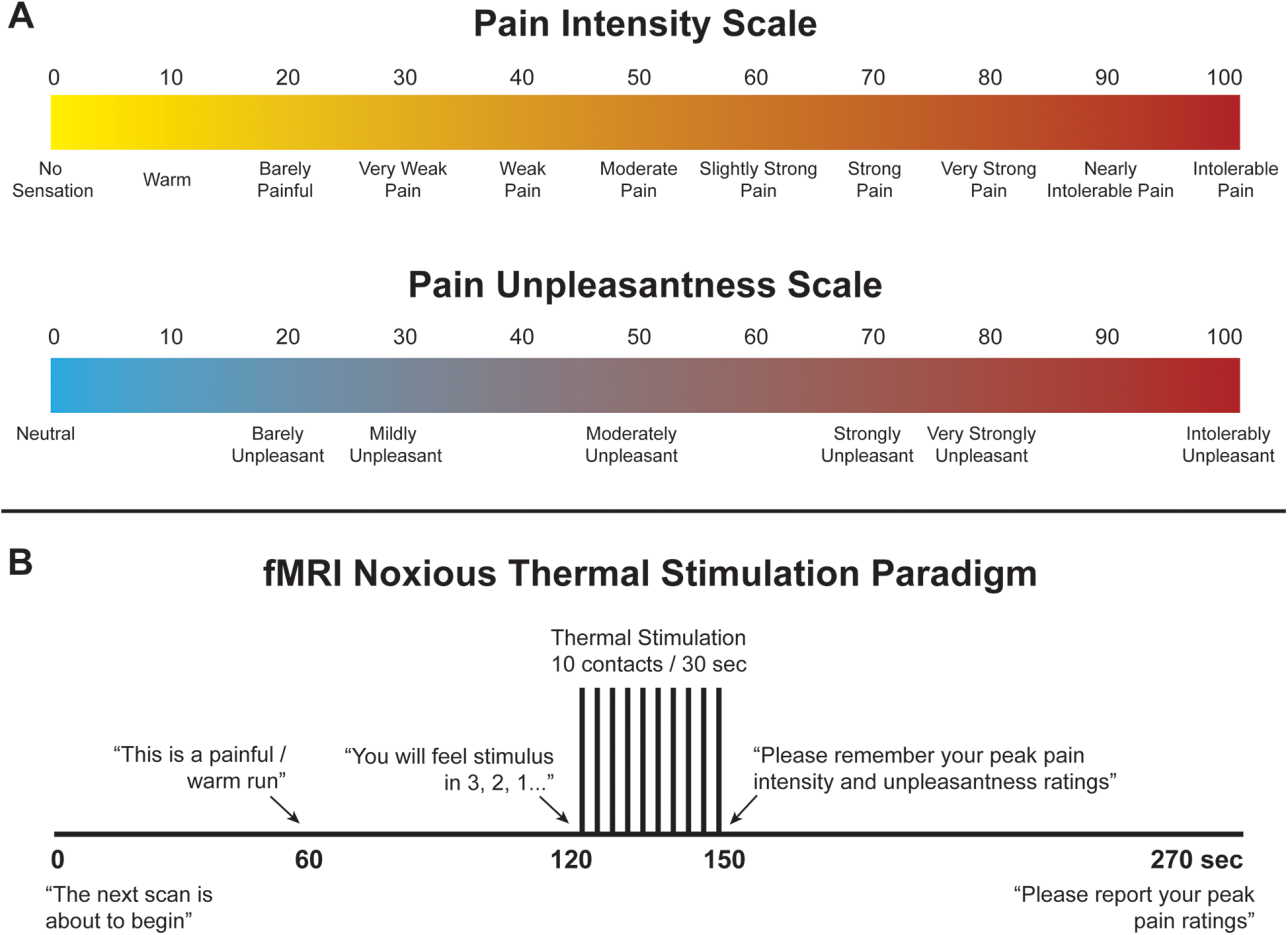
**(A)** Numerical pain scales for intensity and unpleasantness. **(B)** Noxious thermal stimulation paradigm used during training and functional scans.

Immediately prior to imaging, participants underwent a 45-minute stimulus and paradigm training session in a “sham” MRI lab within the Queen's University MRI Facility. The purpose of training was to familiarize participants with the study paradigm, including pain rating scales, the noxious thermal stimulus and timing of stimulation, as well as to ease anxiety and practice laying still to reduce overall bulk motion while in the MRI system. This practice also enabled us to confirm that participants could identify a noxious stimulus as being painful and an innocuous stimulus as being non-painful, using the pain rating scales. Participants were trained to use validated 100-point numerical pain intensity and unpleasantness rating scales (NPS), with verbal descriptors at intervals of 10 (Figure 1A) (41–43). Participants were encouraged to rate in increments of 5 (i.e., using values such as 45, 50, 55, etc.) to simplify the rating process, and the researcher continuously checked each rating with the participant to ensure that they were accurately implementing the scales. They were informed that pain intensity describes more of the discriminative aspect of pain whereas unpleasantness describes the emotional/affective component of perceived pain. The ratio of each participants' pain intensity rating to the temperature needed to elicit that pain rating was used as a “normalized pain score”. A higher pain score may indicate that participants who experienced a particular pain rating at a lower temperature are more sensitive to noxious stimuli (perceiving it as being more painful) than those that reported the same pain rating but required a higher temperature to produce it. This method was used to standardize our pain measures as each participant was individually calibrated to an experimental stimulus temperature which would produce a target pain rating of approximately 50 on a 0–100 scale, as described below.

To elicit acute experimental pain, noxious thermal stimulation was applied with an MRI-compatible robotic contact-heat thermal stimulator (RTS-2), which raised and lowered an aluminum thermode to and from the participants' skin via a pneumatic piston. This device was custom-made within our lab. The stimulus was applied to the thenar eminence of the right hand, corresponding to the sixth cervical segment of the spinal cord. The timing and duration of heat-contacts, along with thermode temperature, were under precise control by custom-made software in MATLAB® (Mathworks Inc., Natick, MA). Each test consisted of ten 1.5 s heat contacts with onsets every 3 s, over the span of 30 s to elicit sustained behavioural and neural responses and to avoid habituation of receptors. Participants experienced a variety of temperatures presented in the same order (ranging from 45 °C to 52 °C) and, once familiarized with the stimulus, were individually calibrated to a temperature corresponding to a tolerable average pain rating of 50 intensity units (“Moderate Pain”, Figure 1A). Participants were kept blinded to this objective, as well as to the temperatures used during the tests, to avoid any potential response bias. The upper limit of 52 °C was set to avoid causing damage to the skin. Additionally, participants were instructed to remove their hand from the RTS-2 if their pain ratings ever exceeded 70 intensity units in order to avoid causing distress or very strong pain. Participants were also informed that there would be two conditions applied while they were in the MRI system, a painful condition at the calibrated temperature (“Noxious”), and a warm, non-painful condition at a standard 40°C for all participants, similar to body temperature (“Innocuous”). Once participants were calibrated, they moved on to the next stage of training.

A mock-up of the MRI scanner (sham-MRI) was used to train participants on the stimulation paradigm and timing that they would experience in the MRI, and to familiarize them with the confined environment. This process was also intended to reduce variations in the data that may be caused by anxiety and bulk motion across repeated fMRI acquisitions. Participants were positioned supine in the sham-MRI with a mirror over their eyes to view a rear projection screen showing the pain intensity and unpleasantness scales, and the RTS-2 under their right hand. A simulated version of the Pain fMRI protocol was carried out at the calibrated temperature. Beginning with a baseline period of 120 s of no contact, the stimulus then made 10 contacts to the skin over 30 s, followed by 120 s of baseline (270 s total, Figure 1B). This method of stimulation was chosen to enable comparisons with our previous studies, and because it produces temporal summation of pain while avoiding habituation of nociceptors in the skin, and thus evokes robust BOLD responses (15, 44–46). Participants were instructed to silently rate the intensity and unpleasantness of each contact as they felt them, and to remember only the highest ratings on both scales. The peak ratings of pain intensity and unpleasantness were recorded, and the calibration temperature was confirmed or adjusted based on these ratings. While the task of mentally rating the intensity and unpleasantness of each contact may have influenced the pain or sensations that were experienced, this procedure was common to runs with noxious and innocuous stimuli and is essential to enable comparisons of the participant's experience to the measures obtained with fMRI.

#### 2.2.2 Functional MRI data acquisition

Functional MRI was carried out on a Siemens 3 tesla MRI system (Siemens Magnetom Prisma, Erlangen, Germany). Participants were positioned head-first and supine with foam supports under their knees and surrounding their head to minimize bulk motion during scanning. The peripheral pulse was recorded from all participants with a sensor attached to their left index finger, and they were provided with a squeeze-ball to signal the experimenter in the event of an emergency, or if they did not wish to continue the study. The RTS-2 was positioned at their side, under the palm of the right hand, and foam earplugs were provided to dampen the loud noise from repeated scans. A 64-channel head and neck coil array was used to obtain images of the brain and brainstem, and a mirror positioned above the participants' eyes allowed them to view a rear projection screen which displayed a presentation of prompts and the pain rating scales during each run which were synchronized with the timing of each scan.

Before the onset of an fMRI run, participants were informed that scanning would commence, and at 60 s after the start of the run they were told which condition to expect for that run (i.e., Pain or Innocuous), at 120 s the participant was informed of the impending stimuli and the pain rating scales were displayed during and after the thermal contacts, and finally, at the end of the run, the participant was instructed to remember their peak ratings and wait for the experimenter to ask for them over the two-way intercom (Figure 1B). After setup, participants were instructed to remain as still as possible and wait for the experimenter to announce the beginning of each scan.

Each imaging session began with the acquisition of “localizer” images in three planes to provide a reference for subsequent slice positioning. A sagittal, T1-weighted anatomical scan was acquired to aid in normalization of functional data using a 3D MPRAGE sequence with 1 × 1 × 1 mm^3^ resolution, a repetition time (TR) of 1,760 msec, echo time (TE) of 2.2 msec, inversion time of 900 msec, and flip angle of 8°. A gradient-echo imaging method with echo-planar spatial encoding (GE-EPI) was used with a flip angle of 90°, and BOLD contrast. The 3D volume spanned from the top of the first cervical vertebra to the apex of the skull and was imaged with a TE of 30 msec for optimal T_2_*-weighted BOLD sensitivity in the brain. The TR was set at 3 s per volume, and 90 volumes were recorded to produce a time-series spanning 270 s (4.5 min). Data were acquired in 49 contiguous axial slices, 3 mm thick, with a 192 × 192 mm field of view, and a 64 × 64 matrix, resulting in 3 mm isotropic resolution, with an anterior/posterior phase-encoding direction.

Multiple runs of each condition (Pain and Innocuous) were acquired in a randomly interleaved order and participants were informed of which condition to expect after 60 s of scanning (60 s prior to stimulation). The stimulation paradigm followed the sham-MRI run exactly, beginning with 120 s of baseline scanning, followed by 30 s of 10 heat contacts, and then 120 s of baseline. Participants provided their peak pain intensity and unpleasantness ratings at the end of each run, and these ratings were recorded. In between each run, the MRI operator confirmed that the participant was comfortable and alert before continuing. We aimed to acquire 10 total runs for each participant, 5 Pain and 5 Innocuous, creating a complete dataset with 450 volumes/condition/participant, however 3 participants ended the scanning session with 9 total runs due to technical issues and time-constraints.

### 2.3 Data analysis

#### 2.3.1 Correlational analyses with behavioural scores

To confirm that noxious and innocuous stimulation produced distinct behavioural responses, intensity and unpleasantness ratings and normalized scores were investigated across study conditions using non-parametric Wilcoxon signed-rank tests at a significance threshold of *p* < 0.05. The relationship between questionnaire scores, pain ratings, and normalized pain scores were tested across all individuals using Spearman's rho correlations, with significance inferred at a threshold of *p* < 0.05. Although the sample size is small, we were interested in understanding whether individuals' personal characteristics impact their experience of acute experimental pain.

#### 2.3.2 Data pre-processing

Functional MRI data were pre-processed using Statistical Parametric Mapping software (SPM-12, The Wellcome Centre for Human Neuroimaging, UCL Queen Square Institute of Neurology, London, UK) in MATLAB (version 2021A, MathWorks, Natick, MA, USA). Pre-processing steps included conversion to NIfTI format, co-alignment to correct for bulk motion, slice-timing correction, and spatial normalization to pre-defined anatomical templates from the Montreal Neurological Institute (MNI). The first three time points were excluded to avoid periods of variable T1-weighting. No participants were found to have excessive motion during any of the fMRI runs (>1 mm translation, or >2°of rotation). Images were re-sized to 2 mm cubic voxels prior to normalization for compatibility with the MNI template, and data were “cleaned” to reduce noise by fitting and subtracting signal variations corresponding to the motion parameters determined during co-alignment. Normalizing the data to a common reference also served the purpose of eliminating any effects of participants changing positions between runs, and we were therefore able to combine data across repeated fMRI runs in each person.

Subsequent data analyses focused on characterizing temporal BOLD responses and relationships between regions known or suspected to be involved in pain processing, emotion, and autonomic regulation. To achieve this, we modified a network model that we had previously used in our lab to suit the purposes of this analysis (45, 47). We aimed to identify the relationships between study conditions, individual pain ratings and scores, the timing of the stimulation paradigm (i.e., before and during stimulus application), and personal characteristics (questionnaire scores). For the purposes of prior studies, we had created a combined anatomical template and anatomical region map that spans the brain, brainstem, and spinal cord (47, 48). For this study, the relevant reference images consisted of the MNI152 template, included in SPM12, and anatomical regions maps from the CONN15e software (49). Brainstem regions not included in the CONN15e region map were supplemented based on examples and anatomical descriptions (50–55), and freely shared atlases as described by Pauli et al. (56), Keren et al. (57), and Harvard atlases (http://www.med.harvard.edu/AANLIB/).

#### 2.3.3 Structural equation modeling

**Figure 2:**
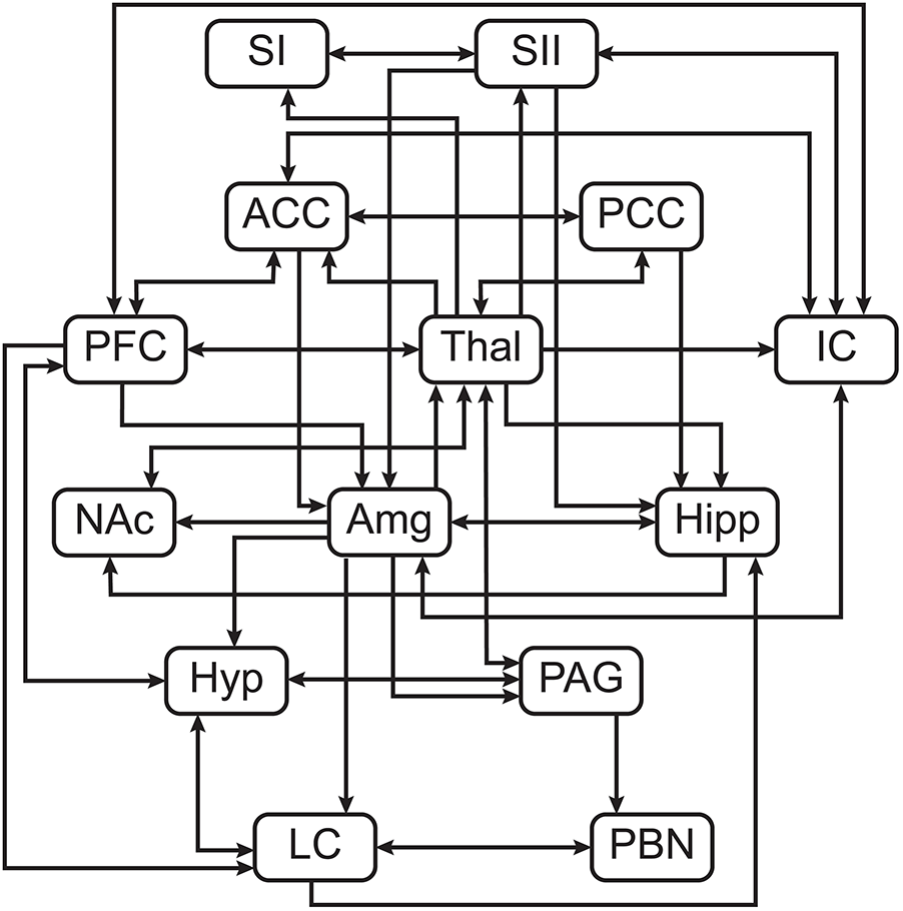
Pre-defined anatomical model of connections between regions of interest.

Structural equation modeling (SEM) is a family of statistical techniques which are used to identify patterns of correlation/covariance among a set of BOLD responses within and across ROIs, and to explain as much variance as possible. This method requires a pre-defined model of directional anatomical connections across the brain and brainstem, based on known neuroanatomy between ROIs (Figure 2), but does not require assumptions about the timing of BOLD signal variations. The network model used in the following analyses includes: ***brain regions***—pre-frontal cortex (PFC), primary somatosensory cortex (SI), secondary somatosensory cortex (SII), anterior cingulate cortex (ACC), posterior cingulate cortex (PCC), insular cortex (IC), thalamus (Thal), amygdala (Amg), hippocampus (Hipp), and nucleus accumbens (NAc); ***midbrain regions***—hypothalamus (Hyp) and periaqueductal gray matter (PAG); ***pontine regions***—locus coeruleus (LC), and parabrachial nucleus (PBN) (52). These areas were chosen to cover a comprehensive array of centres for somatosensation, pain processing and perception, cognitive and emotional processing, and autonomic homeostatic regulation (3, 4, 11, 52, 58–61). Some existing anatomical connections were pruned from this network model to limit the number of comparisons and highlight the most important regions involved in nociceptive regulation and modulation. The same network model was used for all subsequent analyses. Data were averaged across voxels within sub-regions to reduce the number of statistical comparisons to be made and to increase the signal-to-noise ratio over that of single-voxel analyses. Each ROI was functionally divided into seven sub-regions based on time-series characteristics using k-means clustering. Once defined, identical sub-regions were used across the group for both study conditions. This process limits potential bias when dividing each ROI into sub-regions as it assumes that each ROI can have more than one function (15, 62–65). This method has successfully identified robust networks of connectivity across the brain, brainstem, and spinal cord in our previously published work (15, 47, 48, 63–67). For the present study, this method provides the means to investigate coordination of activity across the network of regions, and also enables identification of the sub-regions that best fit the model network. These sub-regions are used as regions-of-interest for subsequent analyses of BOLD responses to the different study conditions.

SEM was carried out by means of a general linear model to calculate linear weighting factors (*β*) which indicate the contribution of each connection to the overall network model, using the time-series data across participants, separately for each condition. If region A receives input signaling from regions B and C, and the BOLD signal time-series responses in these regions are *S_A_*, *S_B_*, and *S_C_* respectively, then: $SA=\beta_{AB}S_{B}+\;\beta_{AC}S_{C}+e_{A}$; where *e_A_* is the residual signal variation that is not explained by the fit (68). The weighting factors were calculated separately for each network component, consisting of a region receiving input (target) with multiple regions providing input (source). However, in order to allow for dynamic variations in connectivity between time periods, the SEM method was applied using data only from selected time periods spanning 45 s (15 volumes/run) before stimulation, and spanning the stimulation period, for each run in each participant. Data from separate runs were combined for each participant. The fitting procedure determines the b values that minimize the residual signal variance. Networks were investigated for every combination of anatomical sub-regions of each ROI to identify the sub-regions that resulted in the best fits to the measured BOLD responses.

The significance of connectivity values (*β*) was determined based on their average values across the group, and the estimated standard errors. Significance was inferred at a family-wise-error Bonferroni corrected *p*_FWE_ < 0.05 which accounted for the total number of network combinations that were tested across combinations of anatomical sub-regions. The network consists of 2–5 source regions for each target region, resulting in between 49 and 16,807 possible network combinations, depending on the target. This statistical approach been validated previously (68). With this process, connections with significant *β* values were identified and used for subsequent second-level analyses. Differences in connectivity across conditions were then tested using paired, two-tailed *t*-tests at a family wise error corrected *p*_FWE_ < 0.05, accounting for the 51 distinct connections in the network. Connectivity weighting factors (*β*) were also analyzed across individuals by computing correlations of *β*-values with individual pain ratings and scores. Ratings from the pain condition were used as a measure of a personal characteristic to test relationships with connectivity values. *R*^2^ values from these correlations were converted to *Z*-scores using the Fisher *Z*-transform with the number of participants. The significance was estimated based on a normal distribution and was inferred at a family-wise error corrected *p*_FWE_ < 0.05, again accounting for the total number of possible network connections, depending on the target region.

#### 2.3.4 Bayesian regression

In order to investigate details of BOLD responses in specific regions-of-interest, a Bayesian regression technique was used to characterize variations in BOLD signal intensity at each time point (i.e., volume) across participants in relation to pain ratings and stimulation temperatures. This analysis was used to identify consistent features of BOLD responses in specific regions which were dependent on individual pain responses. Bayesian regression was applied to the BOLD time-course responses for each sub-region, in each individual, for both conditions (Noxious and Innocuous).

Bayesian regression was applied to each point in the time series, using pain ratings and temperatures as independent variables. The pain ratings and temperatures were first centered so that the average values across all participants were equal to zero, and scaled so that the largest differences from the average were equal to one. BOLD responses in each run were also expressed as the percent signal change from the average for the run. The data were then fit to approximate the consistent BOLD responses (*S*_BOLD_) at the average pain and temperature ratings (*S*_0_), plus linear estimates of the BOLD variations with pain ratings (*S_p_*) and temperature (*S_t_*) (15): $S_{\mathrm{BOLD}}=S_{0\;}+\mathrm{pain}\;S_{p}+\mathrm{temperature}\;S_{t}$. The fitting process therefore enables us to estimate BOLD response patterns (*S*_0_) independent of individual differences in sensitivity or the stimulation temperature used, as well as to identify how the BOLD responses varied systematically across participants with different pain responses. The expected BOLD response for a region can thus be identified at the average stimulation temperature, as being *S*_0_ + *S_p_* at the highest pain rating, and *S*_0—_*S*_p_ at the lowest pain rating. Comparisons of activity were done by linear regression between average time-courses across conditions, for one sub-region at a time, and ranking the slopes of these fits based on their difference from a slope equal to 1 which would indicate perfect correspondence. From this, we chose specific examples of the most similar and least similar activity across conditions, and differences in average time-course activity were determined using point-by-point *t*-tests, corrected for multiple comparisons at a *p*_FWE_ < 0.05.

## 3 Results

### 3.1 Behavioural results

Intensity and unpleasantness ratings and normalized scores were compared across each condition to confirm that the intended effects of noxious and innocuous touch were produced. Perception of stimulus intensity, unpleasantness, and normalized scores (intensity rating/temperature °C) were all significantly different between conditions (Table 1). The calibrated noxious stimulus was consistently rated above 20 NPS units (the threshold for pain), and the innocuous stimulus was consistently rated below 20 NPS units in the non-painful somatosensory range. Scores for the innocuous condition were not used in subsequent analyses in relation to functional MRI data, but rather they were used to confirm that there was a significant difference in stimulus perception.

**Table 1:** Group average values and their standard deviations for intensity, unpleasantness, and normalized scores [intensity rating/temperature (°C)] for Pain and Innocuous conditions. Wilcoxon signed rank tests were performed in each category across conditions (*n* = 19) with significance thresholds of *p* < 0.05, the resulting *Z* and *p* values are recorded.

	Intensity (SD)	Unpleasantness (SD)	Normalized score (SD)
Noxious	43.18 (±8.5)	32.1 (±13.1)	0.85 (±0.17)
Innocuous	9.21 (±2.6)	1.5 (±2.8)	0.23 (±0.07)
*Z*	−3.83	−3.82	17.20–3.82
*p*	<0.001	<0.001	<0.001

Group averages indicated that participants scored around normal or average ranges for all questionnaires and sub-scales (STAI, SDS, BDI-II, and PCS), however scores ranged from low to high, as indicated by the standard deviations from the mean (Table 2). No significant relationships were found between stimulus ratings and questionnaire scores using non-parametric correlational analyses.

**Table 2:** Average scores ± standard deviations (SD) for each questionnaire and their average percentile or range and the Spearman correlation coefficient values (rho) between questionnaire scores and ratings of pain intensity (PI), innocuous intensity (II), pain unpleasantness (PU), innocuous unpleasantness (IU), and normalized pain scores (PS) (*n* = 18).

Questionnaire		Avg. Score ± SD	Percentile/Range	PI (rho)	PU (rho)	PS (rho)	II (rho)	IU (rho)
STAI	Y1	33.6 ± 8.9	46%	0.111	−0.012	0.174	0.113	−0.089
	Y2	37.7 ± 11.2	56%	0.354	0.022	0.380	−0.021	−0.184
SDS		16.5 ± 6.1	Average	−0.358	−0.016	−0.352	0.115	0.200
BDI		8.1 ± 7.0	Average	0.094	−0.118	0.150	−0.150	−0.150
PCS	Total	14.2 ± 8.8	35%	0.019	−0.026	0.023	−0.006	−0.050
	Rumination	6.3 ± 3.5	44.8%	−0.008	0.146	−0.008	0.215	−0.087
	Magnification	3.0 ± 2.5	47.4%	−0.040	−0.096	−0.079	0.005	−0.068
	Helplessness	4.9 ± 4.7	33.5%	0.088	−0.089	0.070	−0.059	−0.080

### 3.2 Functional MRI results

#### 3.2.1 Structural equation modeling

Significant connectivity was found in the periods before and during stimulation across the network in both conditions (i.e., |*β*| > 0), however no significant differences in network connectivity between conditions were found at the group level.

#### 3.2.2 Correlational analyses on SEM results

Differences were found across conditions in terms of which connections were correlated with pain intensity and unpleasantness ratings in the periods before and during stimulation (Table 3). In the noxious condition, connections with *β* values that were correlated with pain intensity ratings included Amygdala → Thalamus and PFC → LC before stimulation, and SII → IC during stimulation. Connections with *β* values that were correlated with pain unpleasantness included NAc → Thalamus before, and PFC → LC during stimulation. In the innocuous condition, correlations between connectivity and pain intensity ratings were seen in connections between PBN → LC before, and NAc → Thalamus during stimulation. Only one connection between PAG → Hypothalamus was correlated with pain unpleasantness ratings during stimulation in the innocuous condition.

**Table 3:** Average connectivity values across the group in each condition that are correlated with pain ratings. *Z*-scores are shown for each correlation, indicating strong relationships between connectivity and pain ratings across the group. Significance is inferred at a family-wise error corrected *p*_FWE_ < 0.05, and respective *T*-values are included for each significant connection (*β*) at the group-level.

Condition	Period	Source	Target	*Z*	*β*	*T*
Correlated with pain intensity						
Noxious	Before	Amygdala	Thalamus	−5.05	0.06 ± 0.04	1.71
		PFC	LC	5.04	0.07 ± 0.1	−0.7
	During	SII	IC	−5.37	0.10 ± 0.03	3.10
Innocuous	Before	PBN	LC	−5.23	−0.29 ± 0.14	−2.12
	During	NAc	Thalamus	5.04	0.22 ± 0.04	6.10
Correlated with pain unpleasantness						
Noxious	Before	NAc	Thalamus	−5.26	0.14 ± 0.04	3.21
	During	PFC	LC	5.07	0.01 ± 0.04	0.19
Innocuous	During	PAG	Hypothalamus	5.11	0.06 ± 0.05	1.16

**Figure 3:**
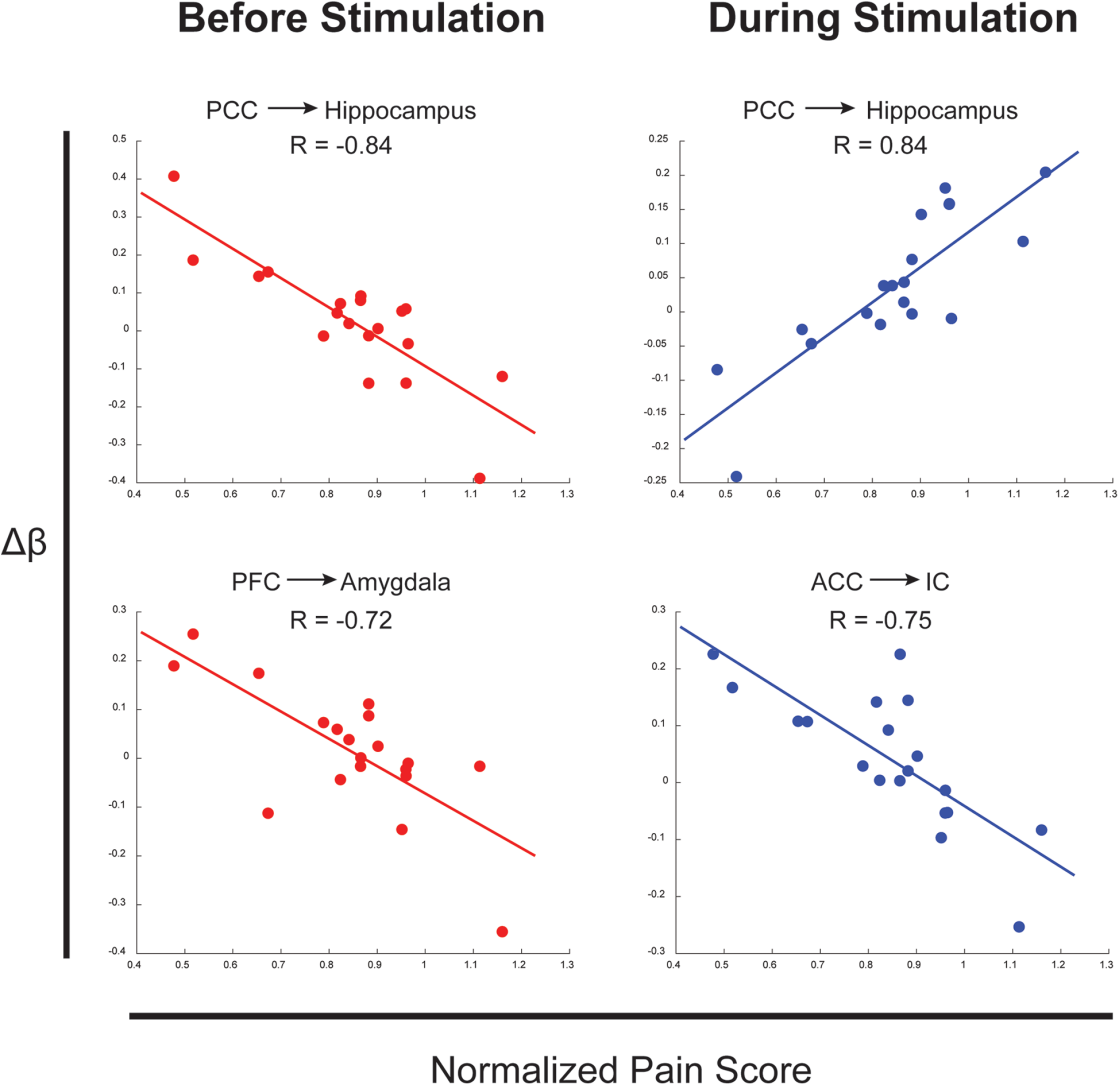
Examples of connections with correlations between Δ*β* across conditions and normalized pain scores. Arrows represent the direction of the connection.

Numerous connections were identified as having strong relationships between the difference in connectivity values across conditions (*Δβ*) and normalized pain scores. These relationships identified effects of individual differences across pain measures and neural connectivity in the brain and brainstem. Examples above a threshold of *R* = 0.7 are shown in Figure 3 for connections between the PCC → Hippocampus, PFC → Amygdala, and ACC → IC in the periods before and during stimulation. These connections all show strong relationships between normalized pain scores and connectivity across the two conditions.

#### 3.2.3 Bayesian regression

**Figure 4:**
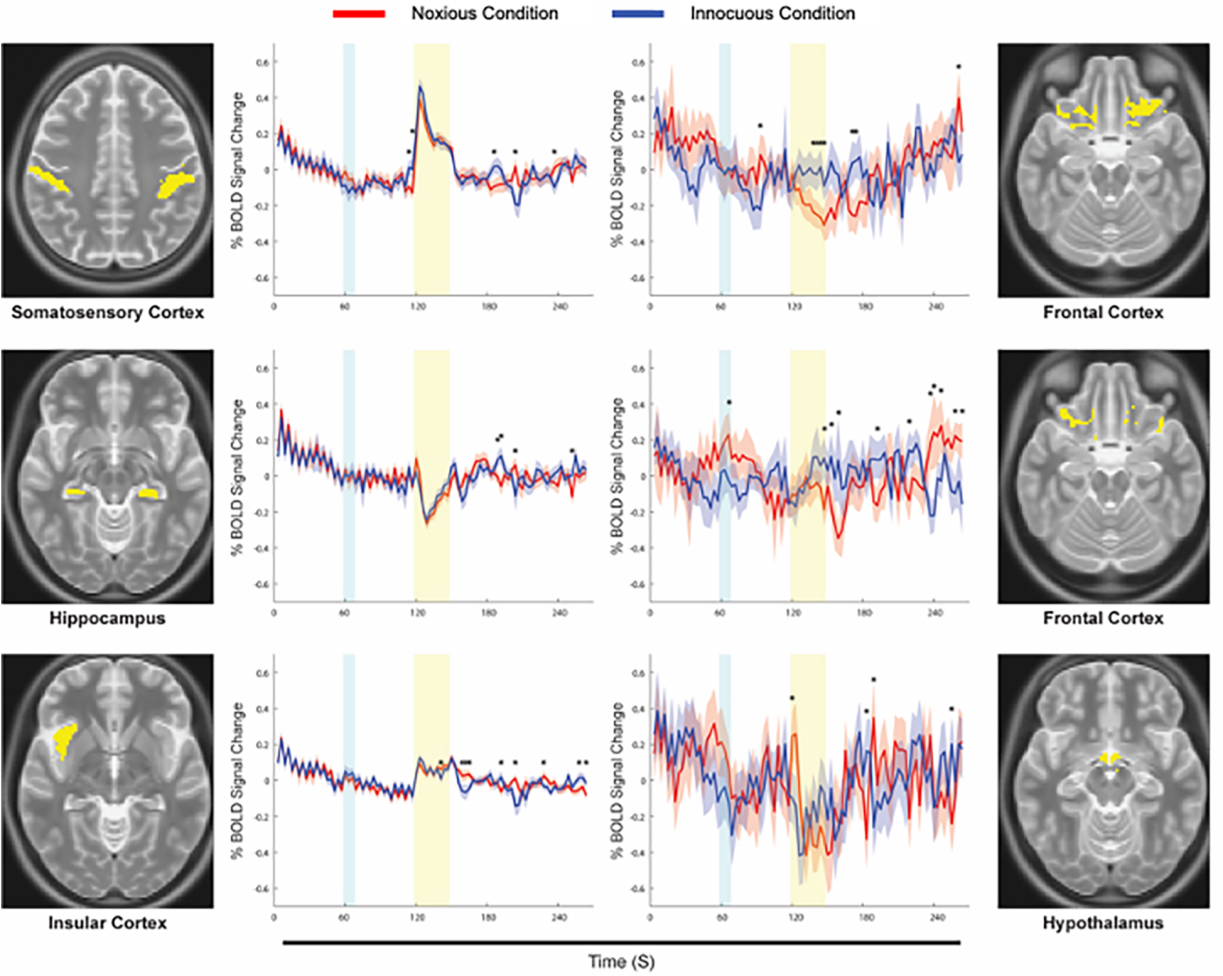
Examples of average time-courses for each condition, calculated via Bayesian regression. The Noxious condition is represented in red, while the Innocuous condition is represented in blue, and standard errors are indicated by the shaded areas around the plotted time-courses. The yellow box indicates the period of stimulation at noxious or innocuous temperatures, and the cyan box indicates when participants were informed of which stimulus to expect in a given run. Anatomical definitions are shown alongside each plot for the corresponding sub-region. Points of significant difference between conditions are indicated with an asterisk (*).

The results of Bayesian regression analyses demonstrated BOLD time-course responses at the average pain intensity rating and temperature for each sub-region and condition. Figure 4 shows BOLD responses from specific sub-regions identified as being the most similar across conditions (left panel), and the least similar (right panel). The strongest agreement across conditions (most similar) were seen primarily in sensory and association regions including SI, SII, ACC, PCC, IC, thalamus, and hippocampus, while the weakest agreements were seen primarily in cognitive, affective, and autonomic regions including the PFC, hypothalamus, amygdala, PBN, and LC. Time-courses were also tested point-by-point for significant differences across conditions, and these are indicated on each plot (*). Note that the BOLD responses and the regions do not depend in any way on the SEM analysis, and the Bayesian regression serves only to provide a linear fit estimate of the time-series values at the average pain rating and stimulus temperature for the group.

## 4 Discussion

In this investigation, we aimed to identify unique neural connectivity underlying the experience of a noxious stimulus which evokes pain compared to an innocuous stimulus, using functional MRI. Significant perceptual differences between noxious and innocuous stimulation were confirmed through behavioural ratings of intensity and unpleasantness, and network connectivity was detected across the brain and brainstem in both conditions. Surprisingly, however, no significant differences in connectivity were found between conditions at the group level, which necessitated further investigation into individual differences to reveal subtleties underlying the different sensory phenomena.

Upon further inquiry, connections were found in both conditions to be significantly correlated with pain intensity and unpleasantness ratings across individuals, and specific connections differed between conditions. Relationships were observed between connectivity strengths in the noxious condition and pain ratings in sensory, cognitive, and emotional regulation regions, while connectivity in the innocuous condition showed a significant relationship with pain ratings in lower midbrain and brainstem regions involved with descending sensory regulation and autonomic processing. Interestingly, the standard errors in connectivity values (*β*) are often quite large compared to the values themselves, indicating a wide range of individual differences in neural responses (Figure 3). The subtleties in connectivity differences across conditions closely relate to variations in pain scores across individuals as a gauge of pain sensitivity and variability. These results contribute to evidence that individual differences in pain perception often relate to one's psychological state (30), and that subtle variations in somatosensory processing of noxious and innocuous stimuli can be differentiated by cognitive, affective, and autonomic feedback (24, 25, 28, 30).

Bayesian regression analyses provided details of average BOLD responses in each of the sub-regions of interest, highlighting the similarities in neural activity across noxious and innocuous somatosensation, as seen in previous investigations (8, 13, 25, 27, 31–33). Predictably, time-course responses in sensory and association regions were the most similar across conditions, while regions involved in cognition, reward, affective, and autonomic processing differed. Responses in “most similar” regions (SI, SII, ACC, PCC, IC, thalamus, and hippocampus) differed significantly in the periods before or after stimulation, while the “least similar” regions (PFC, hypothalamus, amygdala, PBN, and LC) differed across conditions during the stimulation period. This indicates that somatosensory and association regions react to noxious and innocuous stimuli in similar ways, but cognitive and affective regions exert more continuous modulatory control over the experience of different types of stimuli.

These results also show the detail that is captured with our methods, demonstrating coordinated and complex functions, particularly in lower regions of the brainstem. The Bayesian regression technique is sensitive to small changes in signal over time that are consistent across conditions. Both noxious and innocuous stimuli elicit similar low and high frequency trends, with specific differences in certain regions and timepoints. The lack of significant differences in connectivity at the group level and the similarities in time-course responses across conditions are important evidence that the boundary between perception of noxious and innocuous somatosensory stimuli is ambiguous and dependent on an individual's psychological state.

Neural responses to noxious and innocuous somatosensation are closely linked, and difficult to separate at the group level, but subtle differences can be seen in relationships between connectivity and individual behaviours. Pain-specific processing likely occurs to a larger degree in regions associated with descending regulation of pain in the brainstem and spinal cord (7, 19, 48, 65). However, while some results point toward differences being driven by more cognitive, affective, and autonomic regions, the noxious stimulus in this study elicits only moderate pain and is likely not threatening enough to trigger strong fear or emotional responses. Additionally, participants relied on coping strategies based on their own personal pain history, which relate to the wide range of individual differences in responses seen here. Many investigations of pain with fMRI have included additional stimuli to stimulate cognitive or affective modulation of a noxious stimulus such as cognitive tasks (17, 21, 22, 48, 69–73), music (1, 45, 74–77), emotional manipulation (10, 12, 78, 79) etc. These studies have reliably shown group-level differences in neural activity across task and control conditions with noxious stimuli, which may be a more subtle effect than a different physical stimulus. However, along with the present results, this suggests that our cognitive, affective, and interoceptive states have a stronger influence on the way we perceive pain than the somatosensory stimulus itself.

While the results of this study demonstrate a subtle effect of stimulus type across individuals, the limitations of the data should be considered. First, although the noxious stimuli were perceived significantly different than the warm, innocuous stimuli, participants were calibrated to experience a moderate level of pain, which varied from run to run. Therefore, a stronger, more intense noxious stimulus (causing more pain) may elucidate group differences in neural connectivity, however, the upper limit of 52 °C was chosen to avoid over-sensitizing or damaging the skin. Additionally, a larger sample size would provide more statistical power for behavioural analyses alongside questionnaire data and would allow for more in-depth investigation into both group and individual differences in neural responses. Individual differences are an important feature within the complex nature of pain and due to the nature of this data, a large set of detailed results were produced from each analysis that could not be discussed in one text. Future investigations may aim to probe individual differences within this and similar datasets as this would allow for classification of sub-groups and more subtle neural effects within the study sample.

SEM is inherently limited by the number of regions included in the pre-defined anatomical network model, and therefore some possible anatomical connections were omitted. This was done to decrease the necessary computing power and the number of multiple comparisons across sub-regions in the network. Additionally, as each region of interest was functionally divided into sub-regions based on time-course properties, we can only make inferences about connectivity based on the known neuroanatomy. Since time-course responses were found to be most similar in sensory areas across conditions, we believe that differences between noxious and innocuous stimuli may involve brainstem and spinal cord regions in order to continuously modulate incoming pain responses at the level of the spinal cord. Our functional MRI methods were optimized for larger brain regions spanning the somatosensory cortices to the upper pons, limiting the spatial fidelity and BOLD sensitivity in lower brainstem regions, due to challenges with the surrounding anatomy. Therefore, data with finer spatial resolution in brainstem regions is necessary to investigate this effect with greater efficacy in these small regions.

The regions of the brain that were modeled for the analyses in this investigation, such as the prefrontal cortex, insula, and primary and secondary somatosensory regions, all serve multiple functions in an intricately connected network of communication. Therefore, it is difficult to separate specific effects of somatosensation, cognition, emotion, and autonomic modulation of stimuli as many regions coordinate a combination of these functions. Features that can be reliably tracked on time-course data are salient responses to stimuli, and continuous activity that monitor our sensory experience and internal and external environments. Therefore, we believe that investigation of the integration of these effects and differences in patterns of neural activity as they relate to behaviours across individuals are key to understanding the complex nature of pain. The search for biomarkers or signatures of pain may only provide limited benefits when individual differences in these complex processes are so pervasive, therefore a strong contribution of cognitive and affective factors must be considered to account for these individual differences.

## 5 Conclusions

The results of this study contribute to growing evidence that pain is a complex, often misunderstood phenomenon. While we did not uncover specific group-wise differences in brain connectivity between noxious and innocuous heat stimuli, a more subtle effect was seen in the individual differences across the group. These results show that while neural responses to noxious and innocuous stimuli are closely linked, individual differences in pain sensitivity across participants contribute a large degree of the observed BOLD signal variations to these stimulation types. This investigation also supports recent evidence that the cortical regions involved with pain processing are not specific to pain and likely form more of a salience detection system, with regions that span multiple functions for the integration of multi-sensory, cognitive, affective, and autonomic information (22, 35, 36, 80, 81). Future investigations should incorporate an expanded range of stimuli to include strong pain and collect a larger sample to uncover unique effects of noxious stimuli.

## Data Availability

The raw data supporting the conclusions of this article will be made available by the authors on request, without undue reservation.
